# A control framework to optimize public health policies in the course of the COVID-19 pandemic

**DOI:** 10.1038/s41598-021-92636-8

**Published:** 2021-06-28

**Authors:** Igor M. L. Pataro, Juliane F. Oliveira, Marcelo M. Morato, Alan A. S. Amad, Pablo I. P. Ramos, Felipe A. C. Pereira, Mateus S. Silva, Daniel C. P. Jorge, Roberto F. S. Andrade, Mauricio L. Barreto, Marcus Americano da Costa

**Affiliations:** 1 Department of Informatics, University of Almería, Almería, Spain; 2 Center for Data and Knowledge Integration for Health (CIDACS), Gonçalo Moniz Institute, Oswaldo Cruz Foundation (FIOCRUZ), Salvador, Bahia Brazil; 3 Department of Mathematics, Center of Mathematics of the University of Porto (CMUP), Porto, Portugal; 4 Department of Automation and Systems, Federal University of Santa Catarina, Florianópolis, Brazil; 5 College of Engineering, Swansea University, Swansea, Wales, UK; 6 Institute of Physics, University of São Paulo, São Paulo, Brazil; 7 Institute of Physics, Federal University of Bahia, Salvador, Bahia Brazil; 8 Institute of Collective Health, Federal University of Bahia, Salvador, Bahia Brazil; 9 Department of Chemical Engineering, Federal University of Bahia, Salvador, Bahia Brazil

**Keywords:** Epidemiology, Infectious diseases

## Abstract

The SARS-CoV-2 pandemic triggered substantial economic and social disruptions. Mitigation policies varied across countries based on resources, political conditions, and human behavior. In the absence of widespread vaccination able to induce herd immunity, strategies to coexist with the virus while minimizing risks of surges are paramount, which should work in parallel with reopening societies. To support these strategies, we present a predictive control system coupled with a nonlinear model able to optimize the level of policies to stop epidemic growth. We applied this system to study the unfolding of COVID-19 in Bahia, Brazil, also assessing the effects of varying population compliance. We show the importance of finely tuning the levels of enforced measures to achieve SARS-CoV-2 containment, with periodic interventions emerging as an optimal control strategy in the long-term.

## Introduction

Efforts to mitigate and circumscribe the spread of the SARS-CoV-2 virus have so far relied on non-pharmacological interventions (NPIs), set in place by most countries since March 2020, including social distancing, personal protective measures, mass quarantines, and other forms of limiting population movement. While the timely deployment of measures combined with an appropriate breadth of interventions has proven successful in effectively reducing the transmission rates of this virus^1,2^, the socioeconomic impacts caused by extensive lock-downs are notably harsher to lower- and middle-income countries^3,4^, for which rescue spending packages and other bailout plans are unforeseeable. In this sense, finding ways to establish an equilibrium between keeping transmission under control while minimizing damages to the economy and society is highly desirable.

The challenges involved in controlling the SARS-CoV-2 epidemic are many. Using Brazil as an example, it is a very large country that ranks third in the number of reported COVID-19 cases (after the US and India), registering over 8.1 million infections and more than 203,000 deaths^5^. The pandemic spread quickly and, in June 2021, Brazil accounted for more than 17.6 million infections and surpassing 494,000 deaths. First-wave mitigation strategies were largely decentralized, and the majority of governmental interventions occurred by local actions taken by the 26 states and the federal district, and their 5,570 municipalities^6,7^. Still, mitigation efforts were inadequate to keep SARS-CoV-2 transmission under control, and the collapse of health services was described throughout the country, which probably influenced the number of fatal outcomes observed to date^8–12^. Even though in some areas of the country a very high level of infection was reached, such as the city of Manaus with an attack rate of 76%^13^, this was insufficient to prevent new waves of infection, confirming that herd immunity is not a feasible or ethical route to tackle COVID-19^14,15^. Thus, it is a concrete possibility that subsequent epidemic waves could, once again, pose a heavy burden on health services with consequent loss of lives, in line with the recrudescence of transmission observed in many countries, possibly boosted by the resuming of many economic and social activities.

Mathematical models have played a key role in assessing the effectiveness of public health policies and NPIs to contain the spread of SARS-CoV-2, as well as to evaluate the transmission dynamics of COVID-19 and how it is impacted by the movement of people^2,7,16–20^. However, the bridging of model outputs to governmental actions aimed at reducing mobility is limited by the inherent uncertainties surrounding the obtained estimates, interpretation difficulties by policy-makers, and the lack of full understanding of a model’s predictive capabilities and limitations^21^.

Accordingly, control algorithms coupled to epidemiological models provide an intuitive means to derive health policies and NPIs from data^22–25^. By drawing on the availability of widespread mobility traces from cell phones, and building on the premise that circulation of individuals is a chief contributing factor for SARS-CoV-2 transmission^26^, here we report an adaptive Nonlinear Model Predictive Control (NMPC) strategy able to reliably predict an optimal level of governmental interventions to decrease mobility, considering different degrees of social mobility effects, that reduces cases and fatalities and keeps hospitalization requirements below their limits while averting the unnecessary extension of restrictive measures such as lock-downs. We applied the NMPC algorithm to study the disease dynamics in Bahia, the largest and most populous state of Northeast Brazil, with territorial extension comparable to that of France. This framework, however, could be adaptable to deploy in multiple settings and can be particularly useful to other developing nations, which lack the purchasing power of high-income countries to benefit from early vaccine access^27^, and thus will probably have to coexist with the pandemic effects for longer.

## Results

We present our results under the framework of nonlinear disease spread modeling coupled with control theory methods^28^. This strategy is sufficiently general to be applied to different settings and can be replicated with minimal data information requirements, which are available for most other countries, ie. cases, fatalities, and hospitalization occupancy beds. To illustrate the utility of the method, we subdivide the following sections toward studying the transmission dynamics in the state of Bahia, Brazil. Three steps are described: (1) the definition of a compartmental model that expresses the dynamic of cases, fatalities, and health service requirements, taking into account asymptomatic/non-detected cases and social mobility patterns; (2) an extension of this compartmental model by a system identification procedure including optimal gains to improve forecast accuracy; (3) the inclusion of an optimal control algorithm that can reliably direct the lifting, continuation or intensification of NPIs in light of the epidemiological situation (Supplementary Fig. S8). Throughout the text, we use the terms public health policies and NPIs interchangeably to refer to government measures aimed at controlling COVID-19. We were particularly interested in studying policies that resulted in changes to population mobility patterns, since the circulation of (possibly infected) individuals, including a-/oligo-symptomatics, is a key factor sustaining the spread of SARS-CoV-2. We refer to this subset of policies as social distancing measures.

### Enactment of governmental measures and implications on mobility patterns

Considering the large territory and population of Bahia (Supplementary Fig. S1), the COVID-19 dynamics in this state is comparable to that of a whole country. On March 6, the first case was registered in the state, roughly one week after the first confirmed case in the country. By September 15, a total of 285,448 cases had been confirmed, of which 6,040 resulted in deaths. On March 16, the state government established a set of measures to mitigate the transmission. They were implemented and partially eased during the period, primarily targeting specific regions rather than the entire state. Most adopted interventions are related to the restrictions of public events and closure of schools/universities. We note that more detailed information about each government measure is discussed by Jorge et al.^7^. Table 1 depicts the group of measures associated with the guidelines of each public measure, in which the average of all groups indices is defined as the stringency index, $u = 100\times (\text {O}1 + \text {O}2 + \text {O}3 + \text {O}4 + \text {C}1 + \text {C}2)/6$.

**Table 1:** **Categories of governmental measures to mitigate the spread of COVID-19 in Bahia.** Details are described in the work of Jorge et al.^7^.

Measure ID	Guideline	Interval
O1	Cancel public events	[0,1]
O2	Closure of schools/universities	[0,1]
O3	Home-office labor for government employees	[0,1]
O4	Social Isolation	[0,1]
C1	Closure of non-essential activities (business, cultural activities, etc)	[0,1]
C2	Transport lockdown	[0,1]

**Figure 1:**
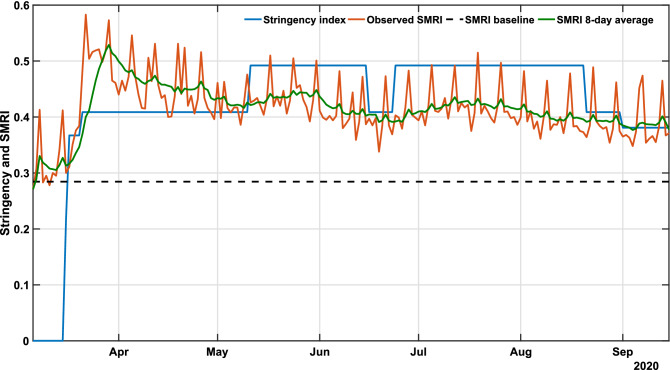
COVID-19 governmental polices and population response. The plot shows the stringency (blue), social mobility reduction (orange) indexes and its 8-day average (green) in Bahia. Raw data from March 6 to September 15, 2020 are shown in this graph. The dashed horizontal line represents the baseline SMRI average between February 1-28, 2020, indicating the level of popular circulation in a pre-COVID period.

Initially, we assessed whether there existed a relationship between the extent of government policies and mobility patterns. For this, the stringency index (*u*), and the social mobility reduction index (SMRI) were used, respectively, as proxies to measure the “strength” of the public policies and the consequent degree of population compliance (Fig. 1). Each policy applied by the government is classified and scored so that the sum of all factors describes the degree of rigidity of government measures. For instance, a decree that allows agglomerations of 100 people contributes to a larger value of the stringency index than a decree that allows agglomerations of 500 people. The definitions of each index and the method used to weigh government measures are detailed in the work of Jorge et al.^7^. Furthermore, the SMRI represents the degree of restriction in the population’s mobility, which means that a higher SMRI represents lower people’s circulation. We observe that the stringency index translates into the SMRI, since one of the purposes of government measures is to increase social distancing, hence increasing the SMRI. The SMRI had a baseline average of 28.5% (February 1-28), which denotes the level of social mobility prior to the identification of COVID-19 in Brazil and before any public policy was applied to reduce social mobility. The SMRI baseline is defined as the minimum level for the control strategy, and it is calculated from the first disclosed data, available in InLoco dataset^29^, and 28 days before the pandemic starts. Supplementary investigations regarding the effect of social distancing policies in Brazil can be found in the work of Barberia et al.^30^. In what followed, six characteristic temporal states were identified: (1) March 6-15: community transmission had been declared in the state, but no governmental measure had been established (stringency $u = 0$; average SMRI $\psi = 30.9\%$; (2) March 16–20: initial measures were set in place ($u = 33.7\%$; average SMRI $\psi = 34.4\%$); (3) March 21-May 10: this period corresponded to the peak population compliance to distancing recommendations ($u = 40.9\%$; average SMRI $\psi = 46.0\%$); (4) May 11-June 15 and June 25-August 20: *u* reached its maximum value of 49.2% (representing the peak of interventions) with average SMRI of $42.2\%$ and $41.2\%$, respectively; (5) June 6–23: population compliance started to decrease with the concurrent reduction in stringency ($u = 40.9\%$; average SMRI $\psi = 39.1\%$); and (6) August 21-September 15: following the peak number of cases, a progressive decrease of both stringency and compliance ensued ($u = 39.1\%$; average SMRI $\psi = 39.0\%$).

### Reproducing the dynamics of COVID-19

To reproduce the transmission dynamics of COVID-19 in Bahia, under the previous described social behavior and governmental interventions, we applied the SEIIHURD+$\psi$ model with all gains $g_{i}$ $=1$ in supplementary equations (1a) to (1h). A sensitivity analysis of the model was performed, allowing identification of key parameters governing the dynamics of this system (detailed in Supplementary Text).

**Figure 2:**
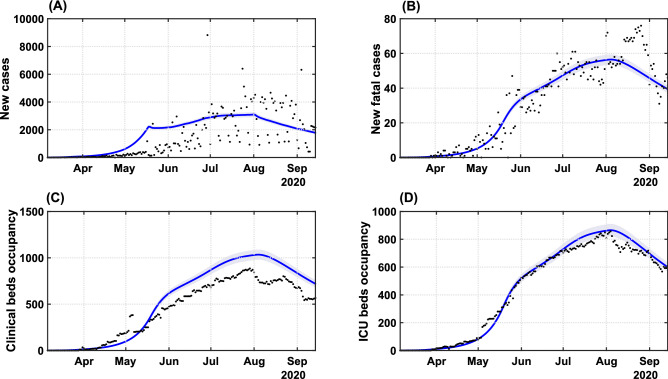
Transmission dynamics of COVID-19 in Bahia. Effects of the implemented interventions, mobility patterns and respective coefficient of determination $R^2$ on the dynamic of (**A**) cases ($R^2 = 0.4684$), (**B**) deaths ($R^2 = 0.8871$), (**C**) clinical hospitalization ($R^2 = 0.7953$) and (**D**) ICU bed requirements ($R^2 = 0.9844$) at the state level. The solid blue lines represent the evolution of the epidemic given by the SEIIHURD+$\psi$ model without gains. The shaded error bands represent 5% of the curve extrapolation margin. The assumed parameter values are shown in Supplementary Table S1. Raw data (black dots) from March 6 to September 15, 2020, are shown in this graph with a daily timescale.

Based on a visually good fit between observed and model-predicted values, the SEIIHURD+$\psi$ model can reproduce the dynamics of COVID-19 with respect to the number of cases, deaths, and clinical hospitalization/ICU bed requirements (Fig. 2). In this simulation, epidemiological parameters of the model were kept fixed (table S2), while the SMRI (given by the registered series $\psi$) and the transmission rate $\beta$ varied in time. The goodness of fit, estimated using $R^2$, varied between 0.4684 (for the number of cases) and 0.9844 (for the ICU requirements). The variability in the official number of new cases is characterized by a seven days period, mainly due to inherent issues in reporting new cases, such as communication delays on non-working days, the high number of counties and the state territorial dimension. Nevertheless, the model identification procedure can identify the series trend and provide a good representation of the pandemic evolution. We next aimed to improve the forecast accuracy of the model, in particular for the prediction of cases, by employing a parameter identification procedure.

### Improving forecasting of cases, deaths, and clinical/ICU occupancy

By drawing on the previous result, considering that the SEIIHURD+$\psi$ model with unitary gains can realistically describe the COVID-19 transmission dynamics, we sought to couple an internal controller capable of predicting optimal social mobility actions. Based on the control theory framework^28^, the internal process in the NMPC algorithm requires an accurate forecast of the pandemic dynamics to formulate predictive control strategies based on a well-posed optimization problem.

Seeking to improve the forecast accuracy of the SEIIHURD+$\psi$ model without compromising the epidemiological parameters, the gains $g_i$ vary every 13 days, which is consistent with the infection dynamics of the SARS-CoV-2 virus (and could provide enough days for the validation tests). As an exception, the transmission parameter $\beta$ may change in time, that is, the gain $g_1$ is allowed to change within the 13 days windows. In particular, when the internal model also has $g_i = 1$, the results refer to a nominal case analysis. The main goal in the identification stage is to adapt the model fit by assuming that the input data series may suffer interference from several factors, such as case under-ascertainment and underreporting, as well as notification delays^31^. In addition, the model parameters may undergo slight variations in the unfolding pandemic due to changes in medical treatments and protocols and the enactment of governmental measures, for instance. Consequently, we conducted an optimization stage to adjust the parameters of the SEIIHURD+$\psi$ model and increase the quality of the predictions, particularly in the short-term, for which an enhanced accuracy benefits the most the results of the control algorithm.

**Figure 3:**
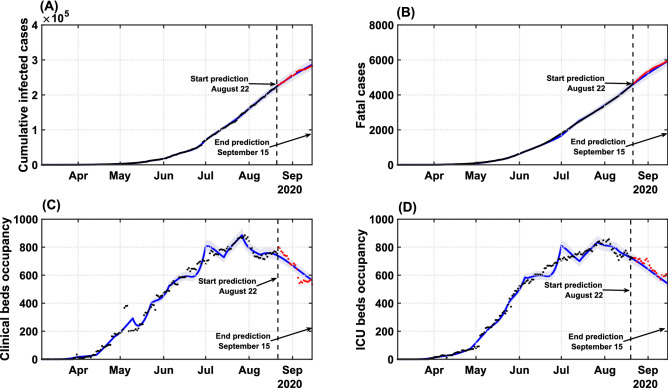
Model validation and forecast of the COVID-19 dynamics in Bahia. Model curves adjusted up to August 22 (blue lines), accounting for the identification procedure for $g_i$ parameters, and respective coefficient of determination $R^2$ for: (**A**) cumulative cases ($R^2 = 0.9998$); (**B**) deaths ($R^2 = 0.9994$); (**C**) clinical hospitalization ($R^2 = 0.9872$) and (**D**) ICU bed requirements ($R^2 = 0.9872$) at the state level. Data beyond the dashed vertical line indicate the predicted values for the epidemiological parameters between August 22 and September 15. The shaded error bands represent 5% of the curve extrapolation margin. The assumed model parameter values are shown in Supplementary Table S4. Raw data (black dots) from March 6 to September 15, 2020, are shown in this graph with a daily timescale.

Figure 3 presents the model validation, adjusted with data up to August 22, 2020, and its forecasts compared with real data for cumulative cases, deaths, clinical and ICU beds occupancy, up to September 15. The results from the identification approach show that, with a proper adjustment of the gains, it is possible to improve the model’s accuracy and offer reliable predictions for up to 25 days in the future. By assessing the coefficient of determination, $R^2$, our results reveal that the optimized model can reproduce the series of cumulative cases ($R^2 = 0.9998$), fatalities ($R^2 = 0.9994$), clinical ($R^2 = 0.9872$) and ICU bed requirements ($R^2 = 0.9872$) with high accuracy (Fig. 3).

The estimated gains are shown in Supplementary Tables S4 and S5. Although we obtained estimates for a total of 13 windows, the variance of the gain series is very small and enough to improve the forecast, indicating that the mathematical model is representative of the underlying epidemic unfolding process. By coupling the prediction capability of the SEIIHURD+$\psi$ model with a control algorithm, an optimal framework for the deployment of governmental interventions that accounts for human mobility patterns can be developed.

### An optimal control guide for public health interventions

Next, we combined the SEIIHURD+$\psi$ model with a predictive control algorithm in order to determine an optimal level of social mobility and governmental measures that would allow a reduction in cases and fatalities and preserve clinical and ICU bed occupancy rates below their limits. We note that the operation of this control algorithm is periodic, in such a way that new public health measures are determined every week, in line with the accurate short-term projections produced by the model. Also, many local governments now rely on multi-phased approaches to ease or increase the level of measures, usually based on objective metrics such as occupation of hospitals, $R_t$, and trajectory dynamics of cases^32–36^. Consequently, our strategy can also support the periodic re-calibration of stringency in phased reopening strategies to minimize the chances of surges.

**Figure 4:**
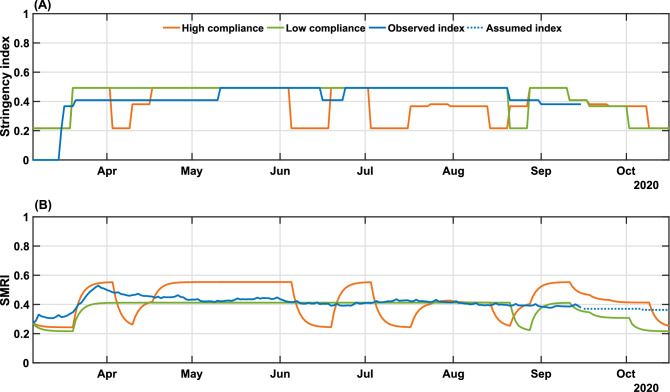
Real and simulated social mobility and governmental interventions in the state of Bahia. Levels of stringency (**A**) and social mobility reduction (**B**) indexes are shown for the high and low compliance scenarios, as well as the actual value of these metrics in the state-level during the period. The observed SMRI values (March 6-September 15) consist of a 8-day moving average. The dotted line in panel (**B**) indicates the assumed values of SMRI as described in Results. The high ($K_\psi = 1.1247$) and low ($K_\psi = 0.8374$) compliance scenarios represent the level of social mobility response related to the applied government measures.

**Figure 5:**
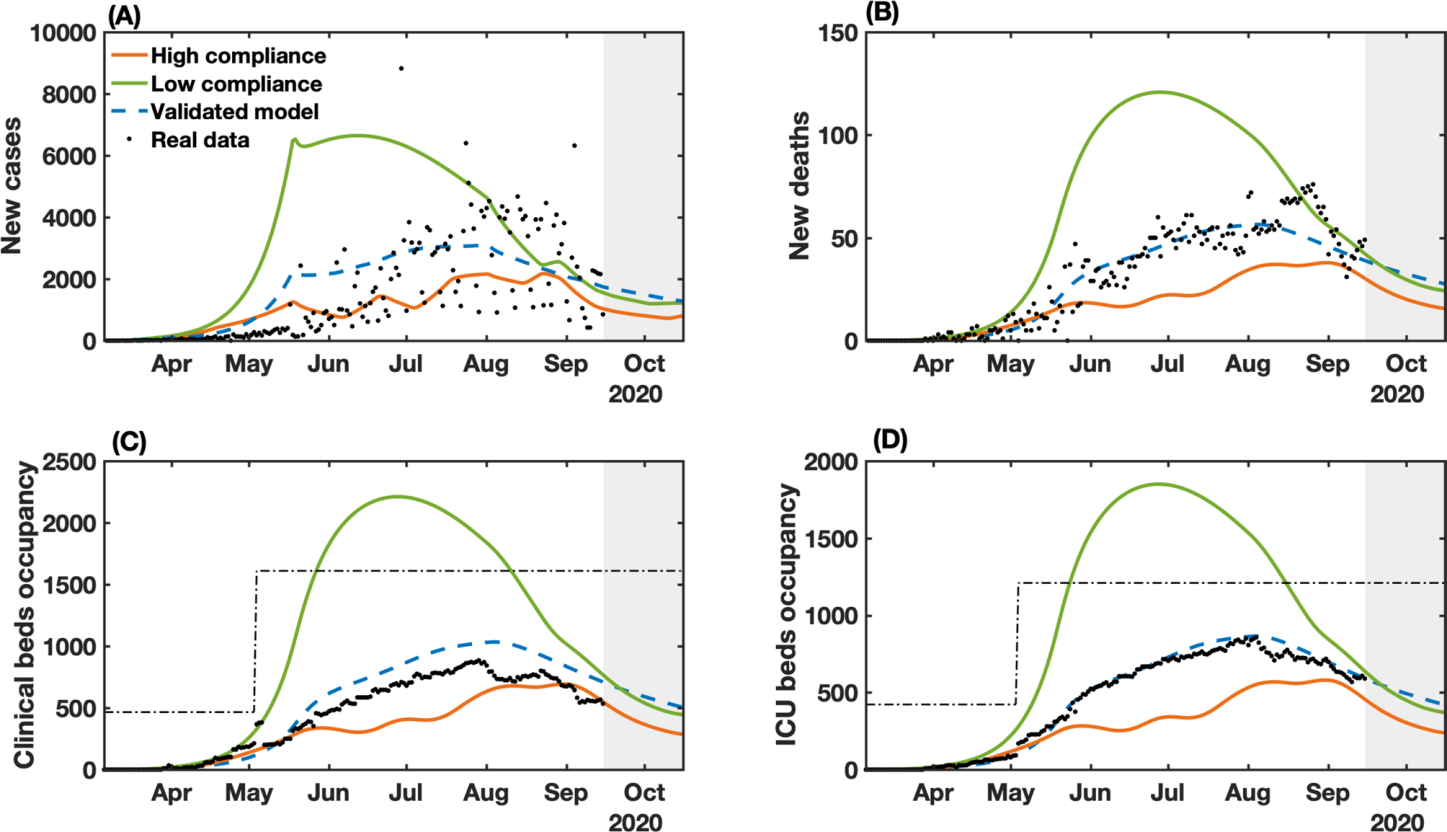
NMPC-controlled simulated epidemic unfolding compared to real-world data in Bahia, Brazil. (**A**) New cases; (**B**) deaths; (**C**) clinical hospitalization and (**D**) ICU bed requirements at the state level. The dashed-blue lines represent the dynamics of the validated model presented in Fig. 2 considering the observed SMRI time series in Fig. 3B. The dashed-dotted lines represent the clinical and ICU bed limits, increased from May on, from 466 to 1610 for clinical beds and from 422 to 1210 for ICU beds. Raw data (black dots) from March 6 to September 15, 2020, are shown in this graph with a daily timescale. The high ($K_\psi = 1.1247$) and low ($K_\psi = 0.8374$) compliance scenarios represent the level of social mobility response related to the applied government measures.

Our control algorithm takes as input the time series of *u* and $\psi$, as presented in Fig. 1. The possible scenarios of people’s response influence the future values of stringency. As shown earlier, governmental measures impact population mobility, including in situations of low population compliance. This scenario is represented in Fig. 1, from early May to the end of August, when a downward trend in the SMRI over time is noted, despite increasing levels of *u*(*t*). Thus, we simulated three scenarios corresponding to high and low degrees of population compliance, translated into high/low mobility patterns, and a third scenario mimicking most closely what actually occurred during the period in terms of population mobility, therefore predicting the required levels of measures to reach epidemic control. For this latter scenario, the series of $\psi (t)$ from March 6 to September 15 was given as input to the model. Since data for the SMRI from September 16 onward was unavailable, as we were projecting into the future, we considered that every three weeks the last SMRI value in the $\psi$ time series would be lessened by 2% until reaching the minimum value measured at the beginning of the pandemic, reasoning that the decrease in the number of cases and deaths would lead to a reduction of the SMRI. We refer to this scenario as “validated model” in Fig. 5, since it uses the actual series of SMRI (truncated on September 16) shown in Fig. 4B.

High and low compliance scenarios were considered by adjusting supplementary equation (11), which provides the mathematical relation between *u* and $\psi$, evaluated on the historical data presented in Fig. 1. We calculated past gains $K_\psi$ by considering the mean value of $\psi$ in each constant period of *u*. Further, we considered only the data series beginning on March 16, when the first interventions were enacted. The calculated gains $K_\psi$ are 1.0208 from March 16-20; 1.1247 from March 21-May 10; 0.8577 from May 11-June 15; 0.9560 from June 6-23; 0.8374 from June 24-August 20; and 0.9974 from August 21-September 15. Therefore, to simulate the control algorithm, we can define the high compliance scenario at $K_\psi = 1.1247$, corresponding to the dynamic at the beginning of the epidemic in the state; and the low compliance scenario at $K_\psi = 0.8374$, which corresponds to the period in which the peak of cases, fatalities, and hospitalizations starts to decline. Therefore, high and low population compliance scenarios are associated with the restriction in social mobility $\psi$ according to the stringency level *u* of public policies. The higher the $K_{\psi }$ value higher will be the social distancing in response to the applied public measures. Note that the SMRI is limited to a maximum of 1 (complete lockdown) and a minimum of 0.2865 (SMRI baseline).

Another important factor to weigh is how stringent governments are willing to be in imposing control policies. While some countries opted for more liberal approaches to tackle COVID-19, such as Sweden, which relied mostly on responsible behavior, others maintained very strict curfews for extended periods (eg. Argentina)^37^. A reasonable goal should be to keep the health care system below its capacity level, albeit our algorithm permits tuning the parameter *Q* to make stringency (*u*(*t*)) more or less flexible, which ultimately impacts economic sectors and social behavior. Therefore, from March 6 to May 15 we assume $Q = 8\cdot 10^4$, from May 16 to August 23 we set $Q = 3\cdot 10^4$ and from August 24 to October 15 $Q = 1\cdot 10^4$. An analysis of this parameter’s variation and its effect on future predictions is given in Supplementary Figs. S5 and S6.

In Fig. 4 we show the results of the NMPC algorithm applied to (re)construct the levels of governmental measures, that varied between 21.62% and 49.21%, enforced from March 6 to September 15, and a predicted scenario from September 16 to October 15, considering the popular compliance settings defined previously. The low and high compliance scenarios are compared with the levels of enacted measures since the start of the pandemic (Fig. 4A).

When we combine low popular compliance with the limited variation of the stringency index between 21.62% and 49.21%, the governmental measures must be maintained most of the time at their peak rate, only allowing for a relaxation at the end of August and up to September 3 until October 15, when the requirements of governmental measures reach their lowest degrees, according to the control algorithm. From the observed data, a stringency value of 49.21% corresponds to a two-third restriction on public events, the closure of all schools and universities, a one-quarter of government employees working from home, a 50% isolation compliance in areas subjected to lockdown, a closure of 28.6% non-essential activities and a one-quarter reduction in transportation (Supplementary Table S3). The low compliance scenario, presented in Fig. 4B, shows that a small variation on the mobility compared to the real-world data could cause drastic changes on the epidemic curves shown in Fig. 5, almost doubling the number of infections and fatalities (by a factor of 1.84 and 1.89, respectively). As a result, the total collapse of the health care system would occur from mid-May until mid-August. The efforts made to expand the number of hospital beds in the state (represented by the increased bed capacity made available from May on, from 466 to 1610 for clinical beds and from 422 to 1210 for ICU beds; Fig. 5C and D) would not be sufficient for the requirements of COVID-19 patients.

In contrast, a more optimistic scenario is obtained with a high population compliance to measures. In Fig. 4A, we observe that when the population has a high level of compliance to measures, the optimal level of stringency can be kept under 40% for most of the time, with the highest values of stringency (49.21%) occurring between April 10 to May 21. To compare with the enforced measures, a 38.10% level of stringency index corresponds to the halting of 66.7% of public events, the closure of all schools and universities, one-quarter of government employees working from home and the closure of 42.8% of non-essential activities^7^. In the high compliance scenario, the control framework yields an improved epidemiological situation compared with the actual scenario. The projection results in an estimated decrease of 38.2% in the number of cases, 37.9% in the number of deaths, and 33% of the maximum occupancy of clinical and ICU beds (Fig. 5). Our results also point that, in highly compliant populations, periodic interventions, i.e. alternating periods of high and low stringency, emerge naturally as the optimal strategy to promote control of the transmission (Fig. 4A). This can be a less dramatic mitigation alternative, which by itself could result in increased population compliance to measures.

The control results from September 16 to October 15 predicts an initial high level of required stringency in both low and high compliance scenarios (Fig. 4), followed by a gradual lifting while keeping the downward trend for the epidemic curves. This initial high-level requirement is intuitive if the aim is to reduce cases as close to the basal level as possible (dashed line in Fig. 5). In fact, in the low compliance scenario the number of cases, fatalities and hospitalizations is higher than the basal level represented, while in the high compliance scenario the transmission was still presenting a slow increase. The proposed control algorithm is able to provide the maximum allowed values of interventions to reduce the number of cases. However, the behavioral response is insufficient to allow a control of the epidemic curves as shown in Fig. 5. An alternative would be to strengthen government measures, in the case of low popular support, as shown in Supplementary Figs. S3 and S4.

Finally, comparing the control framework results with the unfolded local scenario, we note that the proposed control algorithm is able to maintain the clinical and ICU bed occupancy below the thresholds of available beds, reduce the total number of infection and deaths, while keeping approximately the same level of applied measures.

## Discussion

In this work, we introduced a framework for optimizing the required levels of public health policies, that translate into variable social distancing effects, during an unfolding pandemic. This tool combines control theory, parameter identification, and nonlinear dynamic modeling to optimize the level of governmental measures according to human behavior, in terms of mobility patterns, during a pandemic. We extend and validate a compartmental mathematical model^9^ that describes the dynamic of symptomatic and asymptomatic/non-detected cases, deaths, and health service requirements, considering the temporal influence of social mobility. By evaluating the effects of social distancing measures enacted locally, we provide a mathematical relationship between interventions and the degree of compliance of the population, measured by the reduction in people’s mobility. We embedded this model in an adaptive control algorithm that can help set policy targets such as the maintenance, heightening or lifting of NPIs (Fig. 4). The utility of this approach is illustrated by studying the dynamics of COVID-19 in Bahia, Brazil, which offered opportunities for an enhanced control of the epidemic (Fig. 5). However, the method is simple and versatile and can be deployed to the analysis of other infectious diseases in other populations, predicting the level of required measures with accuracy. A benefit of this approach is that the levels of predicted stringency can be fine-tuned to adjust to the fragilities of the targeted population and the government capabilities to implement a measure, which can depend on multiple factors including the availability of local resources and political stability.

We used Bahia, Brazil as a proxy of an area with a large population, limited health infrastructure, and stark socioeconomic inequalities. First, we performed a pre-assessment to reproduce the dynamics of COVID-19 in the state from March 6 to September 15, 2020. By leveraging the real-world levels of locally implemented measures with the observed social mobility patterns in this period, our control algorithm identified optimal time windows where the measures and high level of population response (such as that observed at the beginning of the epidemic in the state) can be applied with different magnitudes. In this scenario, the number of cases, deaths and hospitalizations could have been averted by nearly 64%, and the population would have benefited with more extended periods of relaxation of social distancing measures through the enforcement of periodic interventions, which others have shown lead to improved transmission control^38–40^, while alleviating the multiple deleterious facets of prolonged human confinements. Such an achievement can be strategically combined to reduce the impact on the health system and on the economy. In contrast, our findings highlight the importance of widespread compliance to enforced measures. In a scenario of low popular compliance, governments are forced to increase the level of measures to protect the health care system from collapsing. The results in Bahia show that a low compliance could lead to double the number of cases and deaths, and the accompanying collapse of the healthcare system would be inevitable. This is particularly important when planning measures in less developed countries, where poverty is associated with low education levels and, consequently, difficulties in realizing the importance of actions aimed at controlling spread of the virus^41,42^. More vigorous levels of stringency could further decrease the transmission rates; however, the economic effects of prolonged curfews cannot be ignored.

In practice, our proposal requires some care. In particular, long-term forecasting using mathematical models suffer from inherent uncertainties. A real-world application of our method would require constant re-calibration with newly observed data, which we showed substantially improved the accuracy of the predictions. Also, the underlying implemented model does not account for age structure, heterogeneity in contact patterns or stochasticity. In spite of its simplicity, the short-term predictions are still robust and thus adequate for supporting policy re-calibration in short time windows. Additionally, although we can predict the levels of stringency that should be applied in a region, more studies are warranted to understand how the different categories of intervention, such as the closure of schools, the limits on travels and people’s movements, among other NPIs, influence the reduction of cases, as well as how closely related interventions should be prioritized.

There has been strong interest in trying to define the set of NPIs most effectively capable of delaying the spread of COVID-19^1,2,16,19,43^. As noted, various factors complicate the choice of a particular measure over another: First, there is extensive overlap among commonly enacted NPIs (eg. ban on small- and large-scale gatherings); second, many measures were enacted in parallel or almost successively, hindering the evaluation of their individual effectiveness since they are highly correlated. Haug et al.^2^ performed the most complete analysis of NPI effects to date, systematically measuring the impact on the $R_t$ of COVID-19 of 6,068 individual measures in 79 countries and finding that no single intervention is able to reduce $R_t$ below one, and that measures should be combined and deployed in a timely fashion for maximal efficacy. To avoid the pitfalls associated with having to determine the set of NPIs able to maximally decrease the growth of the epidemic, we instead focused on predicting an optimal level of stringency, that in turn influences the mobility patterns of the population. By plugging this relationship into a control algorithm, we were able to reliably assess the level of measures needed to reach a situation of epidemic control, particularly by averting full occupation of available hospital beds. It would be up to policy-makers to choose a combination of NPIs leading to the required stringency level, as predicted by the controller, since distinct combination of measures can lead to equivalent stringency values^2^. More work is needed to better understand the value of individual NPIs and their optimal use to accomplish control targets.

Deploying this strategy in other settings would require, in addition to the local epidemiological data used during the calibration of part of the model’s parameters^28^, more details of the population’s compliance to measures, as our results point that the level of compliance can markedly influence the dynamics of COVID-19 spread, and these may vary by factors such as education level and degree of individual freedom across countries. Consequently, identical levels of stringency may evoke different behavioral responses according to the compliance of each individual and their emerging collective attitudes–ie. people’s actions, including health behavior, are subject to multiple psychological factors and motivations^44,45^, herein modeled as high and low compliant populations. However, the compartmental model that serves as the basis for the dynamics of contagion is general enough to be readily used in other regions, while also being adaptable to other disease domains.

## Methods

### Data collection

We collected epidemiological data of COVID-19 in Bahia, a state with 14.8 million population in the Northeast of Brazil, for the purpose of applying the model developed here. The data comprise the daily number of registered cases and deaths from COVID-19, as well as the daily number of clinical hospitalization and ICU requirements, obtained from the Secretary of Health of Bahia from March 6 to September 15, 2020. Social mobility is represented by the data from “InLoco”, a Brazilian technology start-up, which is available at http://mapabrasileirodacovid.inloco.com.br^29^. The mobility is measured according to the population behavior constructed from anonymous geo-movement information extracted from 60 million mobile devices throughout the country. The used metric, referred to in the manuscript as the social mobility reduction index (SMRI), is defined in the interval 0–100%. It measures the displacement of devices from its self-defined home location, such that the bigger the index, the lower is the population mobility.

In addition to the epidemiological and the social mobility data, we collected all the decrees aiming to mitigate the spread of the disease in the state of Bahia. This dataset resulted from an update to the effort originally described in work of Jorge at al.^7^. Therein, the authors performed an analysis of all state governmental decrees applied in each Brazilian state, deriving an index to measure the stringency of COVID-19-associated interventions adapted to the Brazilian context, originally reported in^46^. The index evaluate measures related to the Cancellation of public events (O1), Closure of schools/universities (O2), Home-office for governmental employees (O3), Isolation of groups or the whole population (O4), Closure of non-essential businesses and public activities (C1), and Transport lock (C2). As presented by Jorge at al.^7^, the total stringency index is a combination of the evaluation of these measures. The index varies from 0 to 1 meaning that the lower the index, the lower the level of governmental measures to mitigate the spread of the disease in the region.

### Model design

The SEIIHURD model considered here^9^ describes the dynamics of a population divided into compartments as susceptible (*S*), exposed (*E*), asymptomatic/non-detected infections $(I_{a})$ and symptomatic (reported) infections ($I_{s}$). The reported infections may present mild to severe symptoms, thus a proportion of them may require hospitalizations (clinical beds), (*H*), or intensive care unity (ICU) admission (*U*). After the infectious period, individuals may recover (*R*) from the disease. A more severe outcome results in death (*D*) due to COVID-19, after passing for a period of hospitalization or ICU. In this model the transmission is not affected by individuals in *H* and *U* compartments. Also, based on the results presented by Oliveira et al.^9^, we also consider a flux of patients between the *H* and *U* compartments, modeling the condition that patients admitted to a clinical ward may worsen their condition and require an ICU bed; conversely, patients in intensive care may need clinical care prior to discharge and recovery.

We added new definitions to the SEIIHURD model to consider influences on the dynamics due to human behavior and healthcare improvements, by allowing the variation of some gains over time. The resulting model SEIIHURD+$\psi$ is described by the systems of equations (1) below:

1a $$\begin{aligned} \frac{dS}{dt} =&\frac{- g_1(t)\beta (1-\psi (t))S(I_s+g_{11}(t)\delta I_a)}{N} \end{aligned}$$

1b $$\begin{aligned} \frac{dE}{dt} =&\frac{g_1(t)\beta (1-\psi (t))S(I_s+g_{11}(t)\delta I_a)}{N} - \kappa E\end{aligned}$$

1c $$\begin{aligned} \frac{dI_a}{dt} =&(1 - g_7(t) p ) \kappa E - \gamma _a I_a\end{aligned}$$

1d $$\begin{aligned} \frac{dI_s}{dt} =&g_7(t) p \kappa E - \gamma _s I_s\end{aligned}$$

1e $$\begin{aligned} \frac{dH}{dt} =&g_2(t) h g_3(t) \xi \gamma _s I_s + (1-g_4(t) \mu _U+ g_8(t)\omega _U g_4(t) \mu _U) g_5(t) \gamma _U U-g_6(t) \gamma _H H\end{aligned}$$

1f $$\begin{aligned} \frac{dU}{dt} =&g_2(t) h (1-g_3(t) \xi ) \gamma _s I_s + g_9(t) \omega _H g_6(t) \gamma _H H-g_5(t) \gamma _U U\end{aligned}$$

1g $$\begin{aligned} \frac{dR}{dt} =&\gamma _a I_a + (1-g_2(t) h \gamma _s I_s+(1-g_{10}(t)\mu _H)(1-g_9(t)\omega _H)g_6(t)\gamma _HH\end{aligned}$$

1h $$\begin{aligned} \frac{dD}{dt} =&(1-g_9(t)\omega _H)g_{10}(t)\mu _H g_6(t)\gamma _HH+(1-g_8(t)\omega _U)g_4(t)\mu _U g_5(t)\gamma _UU \end{aligned}$$

Specifications of the epidemiological parameters are given in Supplementary Table S5. The temporal series $\psi$ accounting for social mobility patterns is given from the InLoco dataset previously described.

In order to improve the predictions that will dictate control measures, multiplicative gain factors, $g_{i}$’s, are introduced into some terms of the original SEIIHURD model. In this context, the factors $g_{1}, \ldots , g_{11}$ are used to account for temporal uncertainties in the parameters $\beta , h, \xi , \gamma _H, \gamma _U, \mu _H, \mu _U, \omega _H, \omega _U, \delta$ and *p* according to the epidemic data which are read continuously by the control system. The uncertainties upon these parameters may appear over time due to different reasons during the course of pandemic, from noises and errors in the reported data to medical treatments improvements, equipment and medical supplies, and screening and testing measures. As a result, the internal adaptive control model can use these time-varying gains in order to enhance forecasting on the disease’s spread and, thus, improve control performance^22,24^. In contrast, when the terms $g_{i}$ are set to 1, the transmission rate $\beta$ is written in terms of a Heaviside step function as in Oliveira et al.^9^, the equations [1a - 1h] reduce to the SEIIHURD model with social mobility index influencing the exposure to risks of the susceptible population, here defined as SEIIHURD+$\psi$ model with unitary gains.

### Parameter estimation

To carry out our analysis, we consider the literature review presented by Oliveira et al.^9^ to evaluate the key epidemiological parameters $\kappa$, $\gamma _a$ and $\gamma _s$, which are maintained fixed during the current work. The time-varying terms $g_1\beta$, $g_2h$, $g_3\xi$, $g_4\mu _U$, $g_5\gamma _U$, $g_6\gamma _H$, $g_7p$, $g_8\omega _U$, $g_9\omega _H$, $g_{10}\mu _H$ and $g_{11}\delta$ have the epidemiological search interval, shown in Supplementary Table S2.

The estimation is performed by ordinary least square minimization problem in the identification algorithm so that the parameter values adjust the model to the series of cumulative cases (*C*), clinical beds occupancy (*H*), ICU beds occupancy (*U*) and deaths (*D*). In this approach, we define the model parameters as decision variables of an optimization problem, which is formulated to minimize the square error between the model curves and the actual data. The optimization problem is proposed as a constrained nonlinear multivariable function without gradient definition. Therefore, we apply the optimization solver *fmincon*, available in the Optimization Toolbox in MATLAB, developed by Mathworks, in order to find the model parameter values that optimally adjust the model curves to the *C*, *H*, *U*, and *D* series. The absolute error variables terms used in the optimization layer are the following:

2 $$\begin{aligned} Er_{C}(t)& = |C(t) - {\hat{C}}(t,g_1(t),\ldots , g_{11}(t))|, \end{aligned}$$

3 $$\begin{aligned} Er_{H}(t) & = |H(t) - {\hat{H}}(t,g_1(t),\ldots , g_{11}(t))|, \end{aligned}$$

4 $$\begin{aligned} Er_{U}(t) & = |U(t) - {\hat{U}}(t,g_1(t),\ldots , g_{11}(t))|, \end{aligned}$$

5 $$\begin{aligned} Er_{D}(t) & = |D(t) - {\hat{D}}(t,g_1(t),\ldots , g_{11}(t))| \end{aligned}$$

wherein the variables ${\hat{C}}, \ {\hat{H}}, \ {\hat{U}}, \ {\hat{D}}$ are obtained according to the SEIIHURD+$\psi$ model equations [1a - 1h]. The complete optimization problem is formulated as follows:

6 $$\begin{aligned} \displaystyle \underset{g_i, \forall i=1,\dots ,11}{\min } \sum _{t=t_i}^{t=t_f} \left( w_1Er_{C}(t) + w_2Er_H(t) + w_3Er_U(t) + w_4 Er_D(t)\right) \end{aligned}$$

wherein $w_1 = 1, \ w_2 = 5,$ and $w_3 = w_4 = 35$ are pre-selected positive weighting values (tuning parameters), used to normalize the order of magnitude of the total cost and minimize the errors $Er_{C}$, $Er_{H}$, $Er_{U}$ and $Er_D$. The values $t_i$ and $t_f$ define the interval $[t_i, t_f]$ where we want to apply the optimization layer.

When the SEIIHURD+$\psi$ with unitary gains is considered, the time-varying parameter $\beta$ is affected by the population mobility explicit in the model by the series of $\psi$. Thus, the values of $\beta$ are estimated considering the mean value of $\psi$ over the identified interval of variations of $\beta$. Additionally, for the validation of the SEIIHURD+$\psi$ with unitary gains, we do not re-estimate the remaining parameters and they are kept as presented by Oliveira et al.^9^ (Supplementary Table S1).

### Model validation and prediction algorithm

In order to improve the performance of our future prediction, we separated our data into sets: a training set containing the first points, from $t_1$ to $t_{169}$, of each data series and a testing set containing the remaining points of the series going from $t_{170}$ to $t_{194}$, the last point of the data we are using. Within the training set, the data is split into consecutive two-week windows, which are sufficiently large to properly describe typical changes in social behavior. In each of these windows, we applied the optimization procedure defined in equation (6) to obtain gains $g_1\beta$, $g_2h$, $g_3\xi$, $g_4\mu _U$, $g_5\gamma _U$, $g_6\gamma _H$, $g_7p$, $g_8\omega _U$, $g_9\omega _H$, $g_{10}\mu _H$ and $g_{11}\delta$. We used the last estimated optimal values to forecast the remaining 25 days of available data and demonstrate the validity of the forecast.

## The control system

In the course of the COVID-19 pandemic, several measures were employed to overcome the socioeconomic impacts and the spread of SARS-CoV-2. The resulting control policies are passed either as a recommendation or as laws, aiming at reducing mobility, gatherings, and consequently slow the spread of the disease. Jorge et al.^7^ evaluated the combination of restriction measures applied to each Brazilian state at each time *t*, yielding the stringency index of implemented governmental measures. The index varies from 0 to 1 depending on the level of restrictions, where 0 means no COVID-19-specific measure applied and 1 corresponds to the most strengthened restriction, for instance, a full lockdown.

We denote here by *u*(*t*) the value of the control signal (stringency index) at time *t*. The NMPC is formulated considering a prediction horizon $N_{p}$ of three weeks, i.e. $N_p \, = \, 21$ days. However, the optimal control signal is applied only on the first 7 days, being recalculated in the next week. We argue that it is not reasonable to change the measures in smaller periods, which would possibly cause confusion to the population.

Each future control signal *u*(*t*) must be piece-wise constant and increase/decrease in accordance with plausible desired levels of control imposed on social mobility. Accordingly, we consider discrete values for *u*, as given in Supplementary Table S3. Note that the control signal *u*(*t*) lies in the interval $[0.2162, \, 0.4921]$ for the scenario considering the real applied measures. For an hypothetical more rigorous scenario, the control signal lies in the interval $[0.2162, \, 0.6269]$ (Supplementary Figs. S5 and S6). These values were obtained by matching the guidelines enacted in Bahia up to date, as provided by Jorge et al.^7^. The algorithm to evaluate all possible future governmental measure is presented in NMPC algorithm section.

Bearing this in mind, we wanted to estimate what are the minimal measures necessary to guarantee that the number of clinical and ICU beds does not surpass the real capacity available in the state, that is, we seek to maintain:

7 $$\begin{aligned} H(t+j|t)& \le n_{\text {clinical}} \end{aligned}$$

8 $$\begin{aligned} U(t+j|t) & \le n_{\text {ICU,}} \end{aligned}$$

The information about the number of clinical and ICU bed available, denoted by $n_{clinical}$ and $n_{ICU}$, were obtained from the Secretary of Health of Bahia. The values were expanded in the course of the epidemic and it is given by $n_{\text {clinical}}=466$ and $n_{\text {ICU}}=422$ for the period between March 6 and May 4^9^, and $n_{\text {clinical}}=1610$ and $n_{\text {ICU}}=1210$ for the following period.

To maintain the desired levels of hospitalizations requirements given in Equations 7 and 8 , the proposed NMPC algorithm must act in order to ensure the following control objective (trade-off objective):

9 $$\begin{aligned} J_{\text {NMPC}} & = I_s(t_f) + Q u(t), \end{aligned}$$

is obtained, wherein *Q* is the weight tuning parameter used to ponder how much the control signal can vary. In other words, if *Q* is low, *u*(*t*) can vary more freely and achieve higher values, otherwise *u*(*t*) achieves lower values. This trade-off adjustment is used to prioritize the minimization of either the control signal or the number of cases. Moreover, the tuning parameter *Q* can also vary in time to adapt to the priorities and the epidemic situation. In this case, at the beginning, we restrict the control actions, aiming to simulate a more realistic scenario, in accordance to what was achieved by the initial enacted acts. Later, we start to relax the control action in order to reduce even more the number of infections. Lastly, when the number of infections is reducing, we reduce even more the value of *Q*, aiming to avoid subsequent waves of infection.

Of note, the control algorithm can be tuned to adjust to each situation, considering the stage of the pandemic, the level of occupancy of ICU and clinical beds and, mainly, the government’s priorities, with a focus on increasing the restriction and the measures of social distancing or making them more flexible, adopting surveillance policies that work in parallel with the opening of economic activities and social life.

The control optimization process for the horizon of $N_p$ steps is assumed to minimize the number of symptomatic infection cases and also the stringency index measures, defined as follows:

10 $$\begin{aligned} \underset{u(t)}{\min } \, J_{\text {NMPC}}(\cdot ) & = \underset{u_{t}}{\min }\left( I_s(t_f) + Q u(t) \right) , \nonumber \\ \text {s.t.}&\text {SEIIHURD}+\psi \text { Model} \,\,\, \forall \, i \in \, {\mathbb {N}}_{[1, \, N_p]}, \nonumber \\&{\underline{u}} \le u(t+i-1) \le {\overline{u}},\nonumber \\&H(t+i) \le n_{\text {beds}}, \nonumber \\&U(t+i) \le n_{\text {ICU}} \,\text {.} \end{aligned}$$

Given that the NMPC framework offers finitely parametrized social distancing guidelines for the COVID-19 spread, its implementation resides in simulating the validated SEIIHURD+$\psi$ along the prediction horizon with an explicit nonlinear solver and testing it according to all possible control *u*. Thus, the predicted variable $I_s$ at the last sample time $t_f$ of the prediction horizon $N_p$ is used to evaluate the cost function $J_{\text {NMPC}}(\cdot )$. The stringency index that implies in the violation of constraints are neglected. Then, the resulting control value is the one that yields the minimal $J_{\text {NMPC}}(\cdot )$, while abiding to the aforementioned constraints. Finally, the stringency index is applied and the horizon slides forward. This paradigm is explained in the NMPC Algorithm section. We note that this methodology ensures the optimality of the solution *u*(*k*) regarding the control objective $J_{\text {NMPC}}$, as described by Rathai et. al.^47^.

After calculating the optimal control signal, we consider that the population responds with a certain dynamic to governmental measures, as proposed by Morato et. al.^25^. The dynamic response is defined as follows:

11 $$\begin{aligned} \psi (t+1) & = \psi (t) + T_2\varrho _{\psi }\left( K_\psi (t)u(t) - \psi (t)\right) \quad , \end{aligned}$$

wherein $\varrho _{\psi } \, \, = \,\, 0.4 \,\, \mathrm {day^{-1}}$ is a settling time parameter, which is related to the average time the population takes to respond to the enacted social isolation measures and $K_\psi$ is a gain relationship between $\psi$ and *u*. $T_2$ represents the sampling period of the NMPC algorithm of one week.

## Supplementary information

Supplementary material 1 (pdf 1638 KB)

## Data Availability

All data is available within the manuscript or its supplementary materials, as well as in the GitHub code repository at: https://github.com/cidacslab/covid-control-policies.
