# Supplementary material for: A control framework to optimize public health policies in the course of the COVID-19 pandemic
Source: Sci Rep. 2021 Jun 28;11:13403. doi: 10.1038/s41598-021-92636-8 (PMC8239053; doi:10.1038/s41598-021-92636-8)
Supplement: Supplementary file 1 — Supplementary material 1 (pdf 1638 KB) [file 41598_2021_92636_MOESM1_ESM.pdf]

A control framework to optimize public health policies in the course of the COVID-19 pandemic

Igor M L Pataro[1,†*], Juliane F Oliveira[2,3,†*], Marcelo M Morato[4], Alan A S Amad[5], Pablo I P Ramos[2], Felipe A C Pereira[6], Mateus S Silva[7], Daniel C P Jorge[7], Roberto F S Andrade[2,7,**], Maurício L Barreto[2,8,**], Marcus Americano da Costa[9,**]

[1]Department of Informatics, University of Almería, Almería, Spain

[2]Center for Data and Knowledge Integration for Health (CIDACS), Gonçalo Moniz Institute, Oswaldo Cruz Foundation (FIOCRUZ), Salvador, Bahia, Brazil

[3]Center of Mathematics of the University of Porto (CMUP), Department of Mathematics, Porto, Portugal

[4]Department of Automation and Systems, Federal University of Santa Catarina, Florianópolis, Brazil

[5]College of Engineering, Swansea University, Swansea, Wales, United Kingdom

[6]Institute of Physics, University of São Paulo, São Paulo, Brazil

[7]Institute of Physics, Federal University of Bahia, Salvador, Bahia, Brazil

[8]Institute of Collective Health, Federal University of Bahia, Salvador, Bahia, Brazil

[9] Department of Chemical Engineering, Federal University of Bahia, Salvador, Bahia, Brazil

[†]Correspondence: Igor ML Pataro (ilp428@inlumine.ual.es) and Juliane F Oliveira (julianlanzin@gmail.com).

[*]These authors contributed equally to this work.

[**]These authors contributed equally to this work.

June 2, 2021

Abstract

In this document we present the relevant Supplementary Materials accompanying the manuscript “A control framework to optimize social distancing measures in the course of the COVID-19 pandemic” by Pataro, Oliveira *et al.*

Supplementary Figures

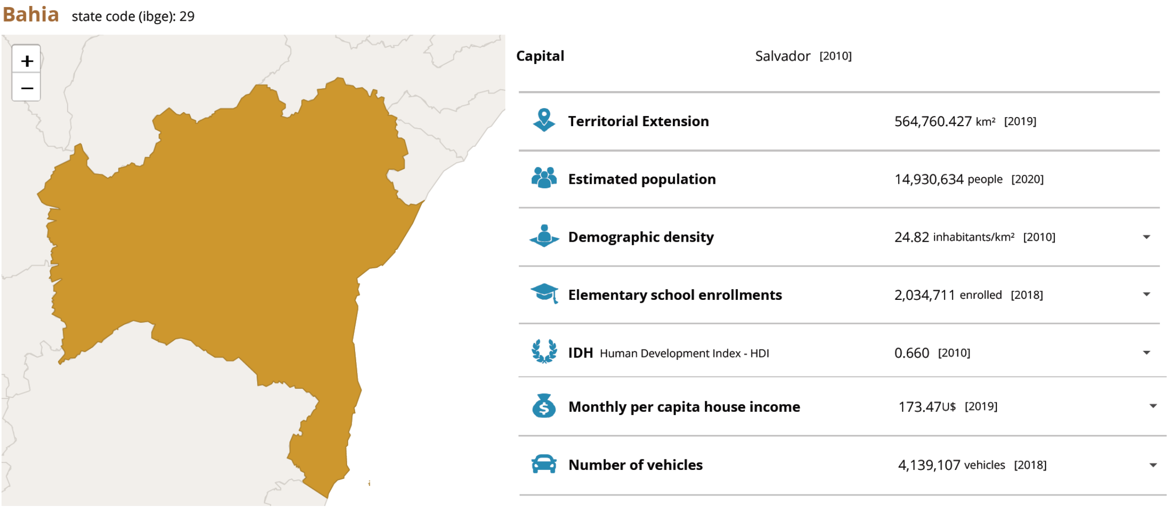

Supplementary Figure 1. A factsheet of Bahia, Brazil, with the main economic and social indicators. Extracted from the Brazilian Institute of Geography and Statistics (IBGE). The exchange rate used to convert Brazilian reais (BRL) to US dollars (USD) was 1 BRL = 0.19 USD (as of Dec 29, 2020).

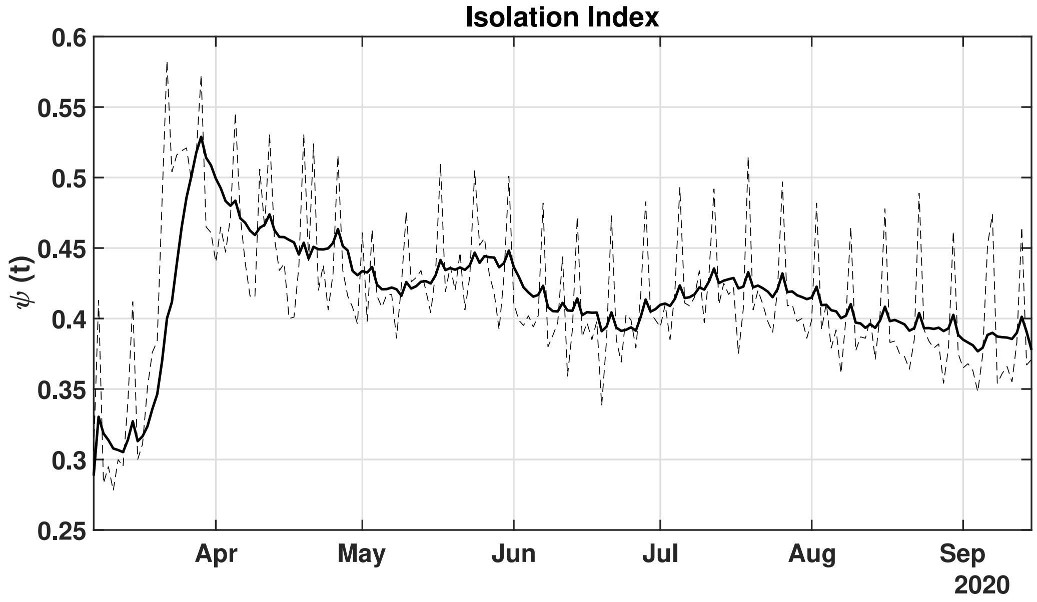

Supplementary Figure 2. Social mobility reduction index. The dashed lines represent the daily percentage of social mobility given by InLoco. The full black line is the moving average mean of 8 days.

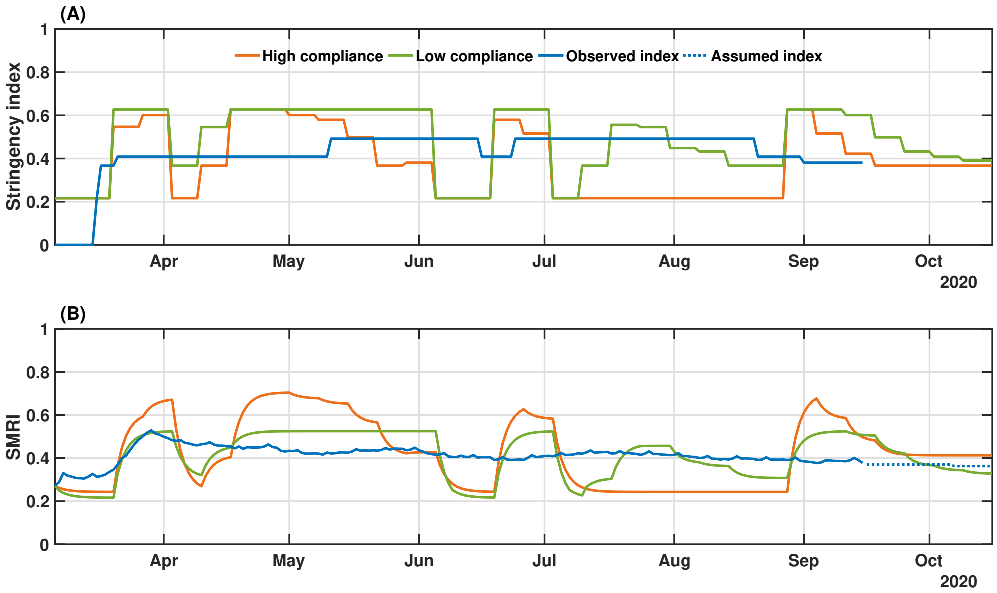

Supplementary Figure 3. Real and simulated social mobility and governmental interventions in the state of Bahia. Levels of stringency (A) and social mobility reduction (B) indexes are shown for the high and low compliance scenarios, as well as the actual value of these metrics in the state-level during the period. The observed SMRI values (March 6-September 15) consist of a 8-day moving average. The dotted line in panel (B) indicates the assumed values of SMRI as described in Results. This scenario represents a hypothetical situation in which the government would have applied 21 decrees with stringency index varying between 0.216 to 0.6269, as described in Table S4.

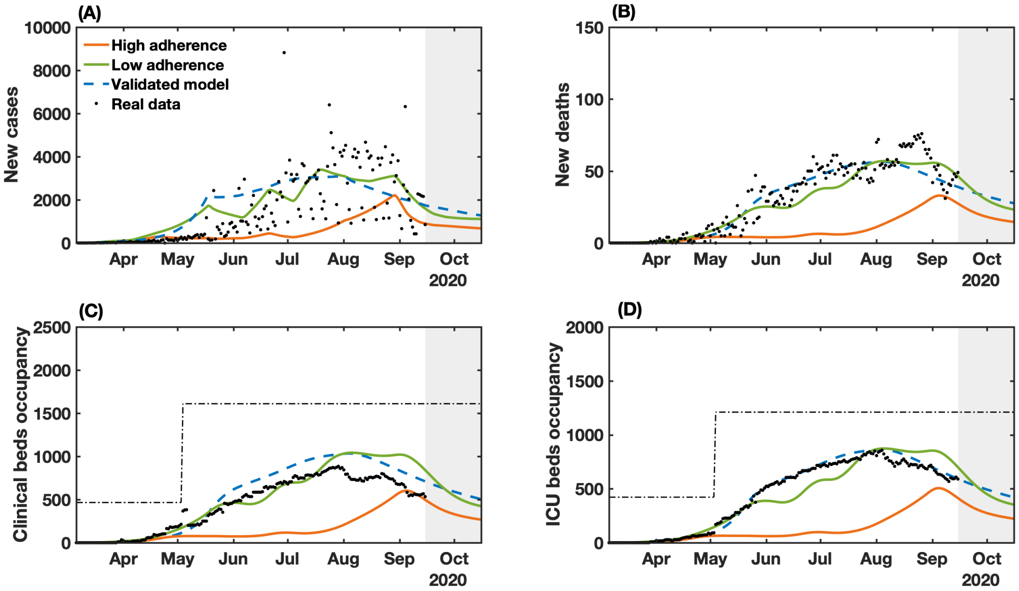

Supplementary Figure 4. Real and simulation-controlled COVID-19 epidemic unfolding in Bahia, Brazil. (A) New cases; (B) deaths; (C) clinical hospitalization and (D) ICU bed requirements at the state level. The dashed-blue lines represent the dynamics of the validated model presented in Fig. 2 considering the observed SMRI time series in Fig. 3B. The dashed-dotted lines represent the clinical and ICU bed limits. Raw data (black dots) from March 6 to September 15, 2020 are shown in this graph. This scenario represent a hypothetical situation in which the government would have applied 21 decrees with stringency index varying between 21.62% and 62.69%, as described in Table S4.

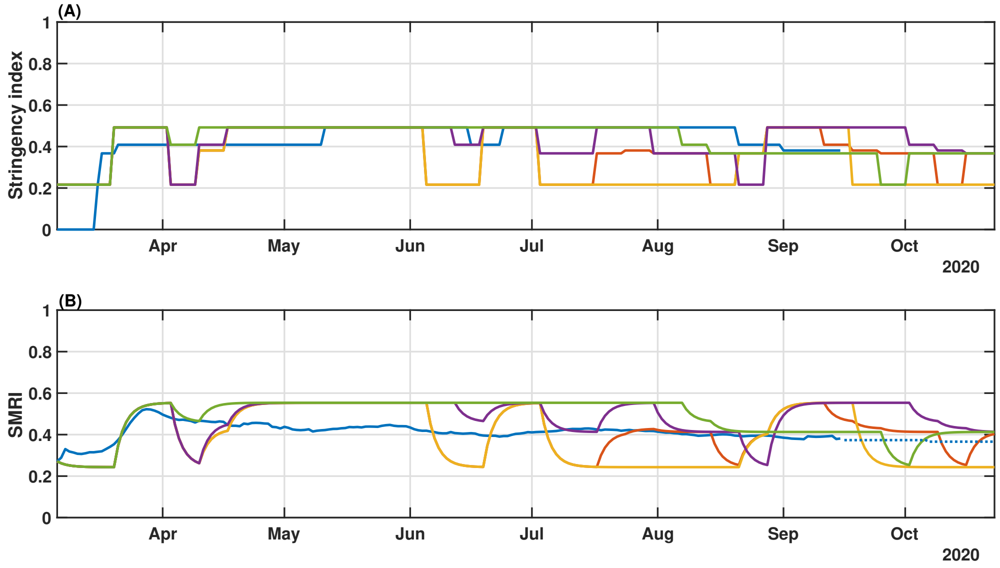

Supplementary Figure 5. Real and simulated social mobility and governmental interventions in the state of Bahia. Levels of stringency (A) and social mobility reduction (B) indexes. The blue-solid line represents the real measured index applied in the state from March 16 to September 15. The dotted line in panel (B) indicates the assumed values of SMRI. This scenario simulates different control tuning with the limited variation of the stringency index between 21.62% and 49.21%, changing the parameter Q to adjust the trade-off between reducing the level of the measures or minimizing the number of cases. Q was adjusted according to the time windows: i) from March 6 to May 15 ii) from May 16 to August 23 and iii) from August 24 to October 15. For the red line Q is $8e4, 3e4, 1e4$. For the yellow line Q is $5e6, 1e6, 2e5$. For the violet line Q is $1e4, 5e3, 1e3$. For the green line Q is $5e3, 1e3, 1e3$.

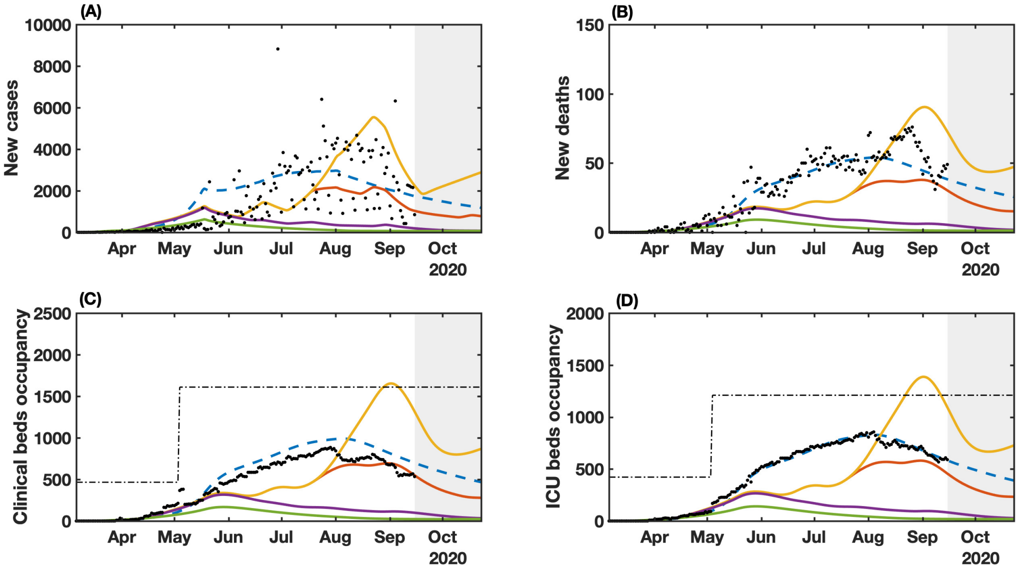

Supplementary Figure 6. Real and simulation-controlled COVID-19 epidemic behavior in Bahia, Brazil. (A) New cases; (B) deaths; (C) clinical hospitalization and (D) ICU bed requirements at the state level. The dashed-blue lines represent the dynamics of the validated model presented in Fig. 2 considering the observed SMRI time series in Fig. 3B. The dashed-dotted lines represent the clinical and ICU bed limits. Raw data (black dots) from March 6 to September 15, 2020 are shown in this graph. This scenario simulates different control tuning with the limited variation of the stringency index between 21.62% and 49.21%, changing the parameter Q to adjust the trade-off between reduce the level of the measures or minimize the number of cases. The Q was adjusted accordingly to the time windows: i) from March 6 to May 15 ii) from May 16 to August 23 and iii) from August 24 to October 15. For the red line Q is $8e4, 3e4, 1e4$. For the yellow line Q is $5e6, 1e6, 2e5$. For the violet line Q is $1e4, 5e3, 1e3$. For the green line Q is $5e3, 1e3, 1e3$.

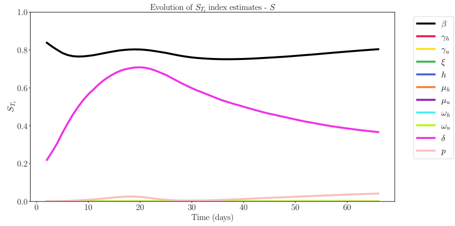

(A) S_{T_i} for S

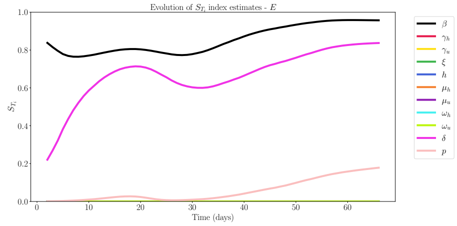

(B) S_{T_i} for E

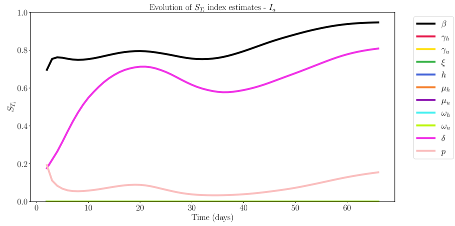

(C) S_{T_i} for I_a

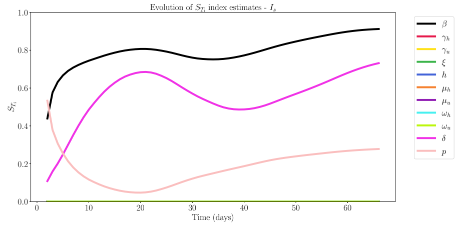

(D) S_{T_i} for I_s

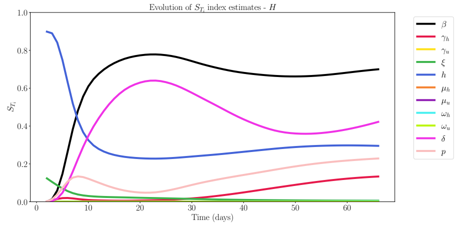

(E) S_{T_i} for H

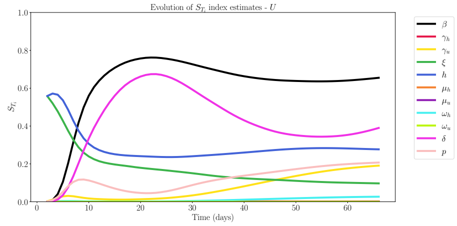

(F) S_{T_i} for U

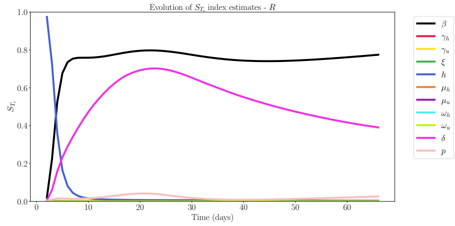

(G) S_{T_i} for R

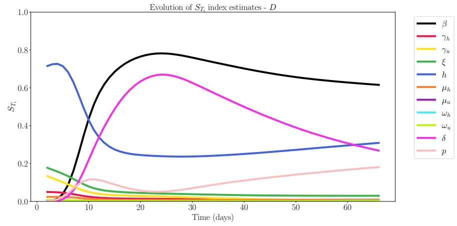

(H) S_{T_i} for D

Supplementary Figure 7. Sensitivity analysis study for S, E, I_a, I_s, U, H, R and D compartments over time. The total effect index S_{T_i} is shown for each evaluated parameter in each compartment of the SEIIHURD model.

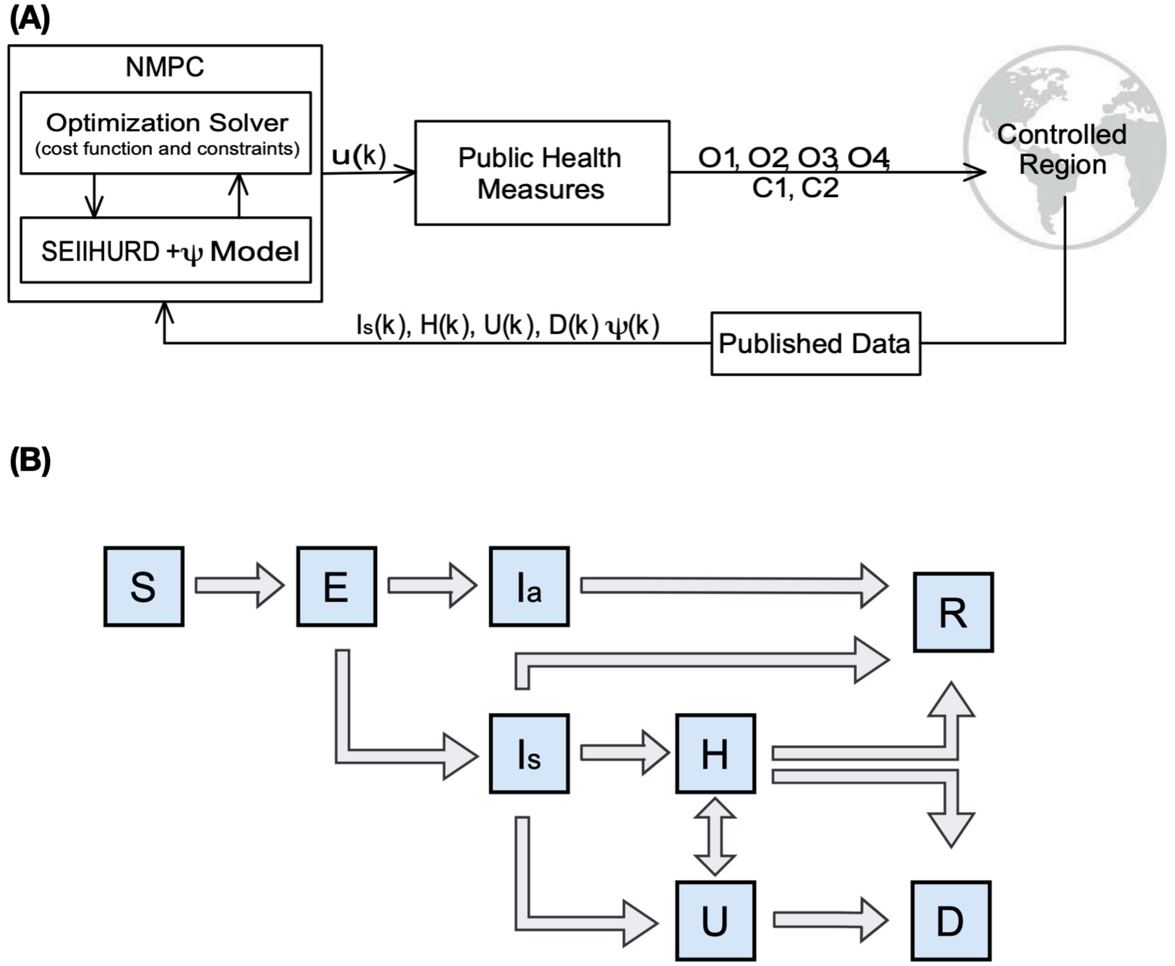

Supplementary Figure 8. (A) Control loop scheme and (B) SEIIHURD compartmental model. The notation k used in (A) defines the discrete sample time in the control algorithm.

Supplementary Tables

Supplementary Table 1. Key epidemiological parameters used in the SEIIHURD+ψ model without gains. Estimates and values for the analysis of the SEIIHURD+ψ without gains with their search interval (when applicable) and respective estimates obtained according to best fit to data up to September 15, 2020 for Bahia. The parameters search intervals were informed by our previous literature mining (*1*).

Parameter	Description	Interval	Fixed	Estimated (95% CI)
β_0	Pre-intervention transmission rate	$[0, 3]$	-	2.13 (2.02-2.24)
β_1	Post-intervention transmission rate	$[0, 3]$	-	1.76 (1.67-1.85)
β_2	Post-intervention transmission rate	$[0, 3]$	-	1.13 (1.07-1.19)
β_3	Post-intervention transmission rate	$[0, 3]$	-	1.00 (0.95-1.05)
t_1	Time of transmission rate change	[March 26th, May 17th]	-	March 26
t_2	Time of transmission rate change	[May 18th, August 1st]	-	May 18
t_3	Time of transmission rate change	[August 1st, September 15th]	-	Aug 1
δ	Asymptomatic/non-detected infectivity factor	[0,0.75]	0.31	-
p	Proportion of latent (E) that proceed to symptomatic infective	$[0.13, 0.5]$	0.2	-
κ	Mean exposed period	$[1/6, 1/3]$	$1/4$	-
γ_a	Mean asymptomatic period	$[1/3.70, 1/3.24]$	$1/3.5$	-
γ_s	Mean symptomatic period	$[1/5, 1/3]$	$1/4$	-
h	Proportion of symptomatic needing hospitalization or ICU	$[0.05, 0.25]$	0.06	-
$1-\xi$	Proportion of symptomatic that proceed to ICU	$[0.01, 0.5]$	0.47	-
γ_H	Mean hospitalization (clinical beds) period	$[1/12, 1/4]$	0.18	-
γ_U	Mean ICU period	$[1/12, 1/3]$	0.13	-
μ_H	Death rate of hospitalized individuals	$[0.1, 0.2]$	0.15	-
μ_U	Death rate of ICU individuals	$[0.4, 0.5]$	0.4	-
ω_H	Proportion of hospitalized that goes to ICU	$[0.1, 0.3]$	0.14	-
ω_U	Proportion of ICU that goes to hospitalization	$[0.1, 0.3]$	0.29	-

Supplementary Table 2. Parameter specifications for the optimizations procedure.

Parameter	Search interval	Initial condition for the optimization problem
$g_1\beta$	[0; 3]	1
g_2h	[0.05; 0.25]	0.06
$g_3\xi$	[0.5; 0.99]	0.53
$g_4\mu_U$	[0.4; 0.5]	0.4
$g_5\gamma_U$	[1/12; 1/3]	0.13
$g_6\gamma_H$	[1/12; 1/4]	0.18
g_7p	[0.13; 0.5]	0.2
$g_8\omega_U$	[0.1; 0.3]	0.29
$g_9\omega_H$	[0.1; 0.3]	0.14
$g_{10}\mu_H$	[0.1; 0.2]	0.15
$g_{11}\delta$	[0.01; 0.75]	0.31

Supplementary Table 3. Control Input evaluation

Control Input Value	Social Distancing Measure ID
0 %	Do nothing
21.62 %	O1 = 0.333, O2 = 0.5, O3 = 0.25, O4 = 0.0, C1 = 0.214 and C2 = 0.00
36.71 %	O1 = 0.667, O2 = 1.0, O3 = 0.25, O4 = 0.0, C1 = 0.286 and C2 = 0.00
38.10 %	O1 = 0.667, O2 = 1.0, O3 = 0.25, O4 = 0.0, C1 = 0.428 and C2 = 0.00
39.00 %	O1 = 0.500, O2 = 1.0, O3 = 0.25, O4 = 0.0, C1 = 0.286 and C2 = 0.25
40.87 %	O1 = 0.667, O2 = 1.0, O3 = 0.25, O4 = 0.0, C1 = 0.286 and C2 = 0.25
41.50 %	O1 = 0.667, O2 = 1.0, O3 = 0.39, O4 = 0.0, C1 = 0.428 and C2 = 0.00
42.23 %	O1 = 0.667, O2 = 1.0, O3 = 0.39, O4 = 0.0, C1 = 0.456 and C2 = 0.01
43.25 %	O1 = 0.667, O2 = 1.0, O3 = 0.25, O4 = 0.0, C1 = 0.428 and C2 = 0.25
44.84 %	O1 = 0.333, O2 = 1.0, O3 = 1.00, O4 = 0.0, C1 = 0.357 and C2 = 0.00
45.90 %	O1 = 0.667, O2 = 1.0, O3 = 0.39, O4 = 0.0, C1 = 0.442 and C2 = 0.25
47.05 %	O1 = 0.667, O2 = 1.0, O3 = 0.39, O4 = 0.0, C1 = 0.511 and C2 = 0.25
49.21 %	O1 = 0.667, O2 = 1.0, O3 = 0.25, O4 = 0.5, C1 = 0.286 and C2 = 0.25
49.80 %	O1 = 0.333, O2 = 1.0, O3 = 1.00, O4 = 0.0, C1 = 0.571 and C2 = 0.08
51.59 %	O1 = 0.667, O2 = 1.0, O3 = 0.25, O4 = 0.5, C1 = 0.428 and C2 = 0.25
54.56 %	O1 = 0.333, O2 = 1.0, O3 = 1.00, O4 = 0.0, C1 = 0.857 and C2 = 0.08
54.69 %	O1 = 0.667, O2 = 1.0, O3 = 0.39, O4 = 0.5, C1 = 0.469 and C2 = 0.25
55.61 %	O1 = 0.667, O2 = 1.0, O3 = 0.39, O4 = 0.5, C1 = 0.525 and C2 = 0.25
57.94 %	O1 = 0.667, O2 = 1.0, O3 = 1.00, O4 = 0.0, C1 = 0.642 and C2 = 0.16
60.12 %	O1 = 0.667, O2 = 1.0, O3 = 1.00, O4 = 0.0, C1 = 0.857 and C2 = 0.08
62.69 %	O1 = 0.667, O2 = 1.0, O3 = 1.00, O4 = 0.0, C1 = 0.928 and C2 = 0.16

Supplementary Table 4. Identified epidemiological parameters used in the SEIIHURD+ψ model with gains. Identified parameter values coupled with its respective gain using the optimization method for a time window of 13 days, starting from March 6, 2020

	Time Window												
Gains	1	2	3	4	5	6	7	8	9	10	11	12	13
$g_1\beta$	0.5486	1.7002	3.0000	2.2302	0.6286	0.9703	1.1252	1.2230	1.2148	0.6290	0.7149	2.5589	0.5730
g_2h	0.0500	0.2500	0.2221	0.2500	0.2500	0.1947	0.1303	0.0568	0.0627	0.0500	0.0847	0.0500	0.0500
$g_3\xi$	0.5000	0.6319	0.5000	0.7181	0.6143	0.8228	0.5000	0.5000	0.5000	0.5107	0.6052	0.5000	0.5000
$g_4\mu_U$	0.5000	0.4000	0.4000	0.4000	0.5000	0.5000	0.5000	0.5000	0.5000	0.5000	0.4000	0.4984	0.5000
$g_5\gamma_U$	0.3333	0.0833	0.1814	0.0932	0.0833	0.0833	0.1001	0.0875	0.0972	0.1631	0.1328	0.1232	0.1521
$g_6\gamma_H$	0.2500	0.0833	0.2500	0.0833	0.1018	0.2500	0.1379	0.1028	0.1169	0.1902	0.2500	0.1923	0.1905
g_7p	0.1300	0.2548	0.1750	0.2732	0.4033	0.5000	0.1969	0.2671	0.2062	0.1300	0.1300	0.1626	0.1796
$g_8\omega_U$	0.1000	0.3000	0.3000	0.3000	0.1000	0.1000	0.1000	0.1000	0.1000	0.1000	0.3000	0.2004	0.1000
$g_9\omega_H$	0.1000	0.3000	0.3000	0.3000	0.3000	0.3000	0.3000	0.3000	0.3000	0.3000	0.1000	0.1000	0.1891
$g_{10}\mu_H$	0.1000	0.1000	0.2000	0.2000	0.2000	0.1020	0.2000	0.2000	0.2000	0.1000	0.1000	0.1000	0.1000
$g_{11}\delta$	0.7500	0.7500	0.0779	0.0100	0.7500	0.7500	0.7500	0.0355	0.7500	0.7500	0.7500	0.0100	0.7500

Supplementary Table 5. Key epidemiological parameters used in the SEIIHURD model.

Parameter	Description
β	Transmission rate
t_i	Time of transmission rate change implicit on the Heaviside step function of β
δ	Asymptomatic/non-detected infectivity factor
p	Proportion of latent (E) that proceed to symptomatic infective
κ	Mean exposed period (days^{-1})
γ_a	Mean asymptomatic period (days^{-1})
γ_s	Mean symptomatic period (days^{-1})
h	Proportion of symptomatic needing hospitalization (clinical beds) or ICU
$1-\xi$	Proportion of symptomatic that proceed to ICU
γ_H	Mean hospitalization (clinical beds) period (days^{-1})
γ_U	Mean ICU period (days^{-1})
μ_H	Death rate of hospitalized individuals
μ_U	Death rate of ICU individuals
ω_H	Proportion of hospitalized that goes to ICU
ω_U	Proportion of ICU that goes to hospitalization

Supplementary Note 1

Parameter sensitivity analysis

Parameter sensitivity analysis

We performed a sensitivity analysis to evaluate the effects of model parameters in the dynamics of I_a, I_s, U, H, R, S, E and D over time. By using an statistical variance-based method, described by Sobol (2001) (*2*), the sensitivity analysis of the SEIIHURD+ψ model considers the following parameter vector

$$\theta := (\kappa, \gamma_a, \gamma_s, \gamma_h, \gamma_u, \mu_u, \xi, h, \mu_h, \omega_h, \omega_u, \delta, p) \in \mathbb{R}^{13}, \tag{1}$$

assuming that its elements are uniformly distributed in proper intervals:

$$\begin{array}{ccc} \beta \sim \mathcal{U}(0,3), & \gamma_H \sim \mathcal{U}(1/12, 1/4), & \gamma_U \sim \mathcal{U}(1/12, 1/3), \\ \mu_U \sim \mathcal{U}(0.4, 0.5), & \xi \sim \mathcal{U}(0.5, 0.99), & h \sim \mathcal{U}(0.05, 0.99), \\ \mu_H \sim \mathcal{U}(0.1, 0.2), & \omega_H \sim \mathcal{U}(0.1, 0.3), & \omega_U \sim \mathcal{U}(0.1, 0.3), \\ \delta \sim \mathcal{U}(0, 1.5), & p \sim \mathcal{U}(0.13, 0.5). & \end{array} \tag{2}$$

To apply this method, we first generated sample values for the input factors shown in Eq. (1) by creating matrices A and B, each with size $N \times n$, where N is the number of samples and $n = 13$ is the number of parameters being analyzed, given by

$$A = \begin{pmatrix} \theta_1^{(A1)} & \theta_2^{(A1)} & \dots & \theta_i^{(A1)} & \dots & \theta_n^{(A1)} \\ \theta_1^{(A2)} & \theta_2^{(A2)} & \dots & \theta_i^{(A2)} & \dots & \theta_n^{(A2)} \\ \vdots & \vdots & \dots & \vdots & \dots & \vdots \\ \theta_1^{(AN)} & \theta_2^{(AN)} & \dots & \theta_i^{(AN)} & \dots & \theta_n^{(AN)} \end{pmatrix} \tag{3}$$

and

$$B = \begin{pmatrix} \theta_1^{(B1)} & \theta_2^{(B1)} & \dots & \theta_i^{(B1)} & \dots & \theta_n^{(B1)} \\ \theta_1^{(B2)} & \theta_2^{(B2)} & \dots & \theta_i^{(B2)} & \dots & \theta_n^{(B2)} \\ \vdots & \vdots & \dots & \vdots & \dots & \vdots \\ \theta_1^{(BN)} & \theta_2^{(BN)} & \dots & \theta_i^{(BN)} & \dots & \theta_n^{(BN)} \end{pmatrix}. \tag{4}$$

We then create n matrices A_B^i, where column i comes from matrix B and all other $n-1$ columns come from matrix A:

$$A_B^i = \begin{pmatrix} \theta_1^{(A1)} & \theta_2^{(A1)} & \dots & \theta_i^{(B1)} & \dots & \theta_n^{(A1)} \\ \theta_1^{(A2)} & \theta_2^{(A2)} & \dots & \theta_i^{(B2)} & \dots & \theta_n^{(A2)} \\ \vdots & \vdots & \dots & \vdots & \dots & \vdots \\ \theta_1^{(AN)} & \theta_2^{(AN)} & \dots & \theta_i^{(BN)} & \dots & \theta_n^{(AN)} \end{pmatrix}$$

In the matrices A, B and A_B^i, each row represents a set of parameter to be used as an input for the model. Numerical simulations are performed, and the output of the sample matrices A, B and A_B^i are stored as the vectors

$$Y_A = \begin{pmatrix} Y\left(A^{(A1)}\right) \\ Y\left(A^{(A2)}\right) \\ \vdots \\ Y\left(A^{(AN)}\right) \end{pmatrix}; \quad Y_B = \begin{pmatrix} Y\left(B^{(B1)}\right) \\ Y\left(B^{(B2)}\right) \\ \vdots \\ Y\left(B^{(BN)}\right) \end{pmatrix}; \quad Y_{A_B^i}. \tag{5}$$

where Y_A, Y_B and $Y_{A_B^i}$ are output vectors.

The final step involves the calculation of the sensitivity indices, using the samples generated during the sampling scheme. We computed the total effect indices, given by

$$S_{T_i} = 1 - \frac{Y_A \cdot Y_B - f^2}{Y_A \cdot Y_A - f^2} \tag{6}$$

where f is defined as

$$f := \frac{1}{N}\sum_{j=1}^{N} Y_A^{(j)}. \tag{7}$$

This index indicates the contribution of the parameter to the output of the model. The importance of each parameter i is proportional to the value of S_{T_i}, meaning that higher S_{T_i} leads to a higher contribution to the model output (*3*). Parameters with higher S_T need a more carefully calibration, as small error during the calibration can lead to larger errors to the predictions generated by the model. The total effect takes into account higher-order interactions among model variables; thus, correlation between variables can also be identified using this method. In addition, we also evaluated the influence of first-order effects, which do not consider interactions among variables, to the model output.

The numerical simulations were performed using the SALib library (*4*). The experiment was conducted generating $N = 15{,}000$ parameter combinations, totaling $195{,}000$ simulations of the model, and the result shows the evolution of the parameters according to S, E, I_a, I_s, U, H, R and D compartments.

The results for the sensitivity analysis of the SEIIHURD+ψ model confirm our previous findings of the sensitivity of the SEIIHURD model, where we showed that the transmission rate, β, and δ, the factor that reduces the infectivity of asymptomatic/non-detected individuals, were among the most influential parameters to every model output during most of the evaluated periods (*1*). This result is recapitulated in every compartment throughout the course of the model dynamics studied herein (Supplementary Fig. 7). In the initial simulation period (up to day 20), the parameters that exert the most impact to the compartments related to severe disease (H, U) and fatalities (D), after β and δ, was h (the proportion of symptomatic needing hospitalization or ICU). In U, the parameter ξ also appears to play an important role in the dynamics of critical cases, which is expected as the parameter $1-\xi$ associates to the proportion of hospitalized symptomatic that proceed to ICU (Supplementary Fig. 7E,F,H). The parameter p, the proportion of latent (E) that proceed to symptomatic infective, appears as an important factor governing the dynamics of individuals in I_s, specially at the initial simulation period and at later time points.

In line with our previous findings on the dynamics of the SEIIHURD model system (*1*), now expanded to the SEIIHURD+ψ framework, the sensitivity analysis conducted confirmed the importance of carefully considering the intervals of β, δ, ξ and h, as these parameters represent important determinants of the dynamics of the model. Indeed, we have previously conducted an extensive literature mining of these key parameters to inform their ranges (Supplementary Table 1 and Ref. (*1*)).

Supplementary Note 2

NMPC Algorithm

NMPC Algorithm

Finitely Parametrized NMPC for Social Distancing Guidelines

Initialize: $N(0)$, $S(0)$, $E(0)$, $I_a(0)$, $I_s(0)$, $H(0)$, $U(0)$, $R(0)$ and $D(0)$.
Require: Q, n_I, n_{beds}, n_{ICU}
Loop, every day:

- Step (*i*): "Measure" the available contagion data ($I(k)$, $H(k)$, $U(k)$, $\psi(k)$ and $D(k)$);
- Step (*ii*): **Loop every week**:
  - Step (a): **For each control sequence** j:
    - Step (1): Build the control vector $\mathcal{U}_k$
    - Step (2): Explicitly simulate the future sequence of the SEIIHURD variables;
    - Step (3): Evaluate if constraints are respected. If they are not, end, else, compute the cost function $J_{\text{NMPC}}(\cdot)$ value.
  - **end**
  - Step (b): Choose the optimal control value $\mathcal{U}_k$ that corresponds to the smallest $J(\cdot)$.
  - Step (c): Increment k, i.e. $k \leftarrow k + 1$.
- **end**
- Step (*iii*): Apply the local control policy $u(k)$.
- Step (*iv*): Simulate the SEIIHURD+ψ model.
- Step (*v*): Increment k, i.e. $k \leftarrow k + 1$.

end

Supplementary References

1. J. Oliveira, D. Jorge, R. Veiga, M. Rodrigues, M. Torquato, N. Silva, R. Fiaccone, L. Cardim, F. Pereira, C. Castro, A. Paiva, A. Amad, E. Lima, D. S Souza, S. Pinho, P. Ramos, R. Andrade, Mathematical modeling of COVID-19 in 14.8 million individuals in Bahia, Brazil, *Nature Communications* **12** (2021).

2. I. M. Sobol, Global sensitivity indices for nonlinear mathematical models and their Monte–Carlo estimates, *Mathematics and Computers in Simulation* **55**, 271 (2001).

3. A. Saltelli, M. Ratto, T. Andres, F. Campolongo, J. Cariboni, D. Gatelli, M. Saisana, S. Tarantola, *Global Sensitivity Analysis: The Primer* (John Wiley & Sons, 2008).

4. J. Herman, W. Usher, SALib: An open-source python library for sensitivity analysis, *The Journal of Open Source Software* **2** (2017).
